# The Prevalence of Cardiovascular Risk Factors among Polish Soldiers: The Results from the MIL-SCORE Program

**DOI:** 10.1155/2020/3973526

**Published:** 2020-05-18

**Authors:** Grzegorz Gielerak, Paweł Krzesiński, Katarzyna Piotrowicz, Piotr Murawski, Andrzej Skrobowski, Adam Stańczyk, Agata Galas, Beata Uziębło-Życzkowska, Anna Kaźmierczak-Dziuk, Joanna Maksimczuk, Sylwia Miernik-Podleśko, Monika Grzęda, Emilia Sopolińska, Marek Kiliszek, Lidia Wojda

**Affiliations:** Department of Cardiology and Internal Medicine, Military Institute of Medicine, Warsaw, Poland

## Abstract

The MIL-SCORE (Equalization of Accessibility to Cardiology Prophylaxis and Care for Professional Soldiers) program was designed to assess the prevalence and management of cardiovascular risk factors in a population of Polish soldiers. We aimed to describe the prevalence of cardiovascular risk factors in the MIL-SCORE population with respect to age. This observational cross-sectional study enrolled 6440 soldiers (97% male) who underwent a medical history, physical examination, and laboratory tests to assess cardiovascular risk. Almost half of the recruited soldiers were past or current smokers (46%). A sedentary lifestyle was reported in almost one-third of those over 40 years of age. The prevalence of hypertension in a subgroup over 50 years of age was almost 45%. However, the percentage of unsatisfactory blood pressure control was higher among soldiers below 40 years of age. The prevalence of overweight and obese soldiers increased with age and reached 58% and 27%, respectively, in those over 50 years of age. Total cholesterol was increased in over one-half of subjects, and the prevalence of abnormal low-density lipoprotein cholesterol was even higher (60%). Triglycerides were increased in 36% of soldiers, and low high-density lipoprotein cholesterol and hyperglycemia were reported in 13% and 16% of soldiers, respectively. In the >50 years of age subgroup, high and very high cardiovascular risk scores were observed in almost one-third of soldiers. The relative risk assessed in younger subgroups was moderate or high. The results from the MIL-SCORE program suggest that Polish soldiers have multiple cardiovascular risk factors and mirror trends seen in the general population. Preventive programs aimed at early cardiovascular risk assessment and modification are strongly needed in this population.

## 1. Introduction

Despite the remarkable progress in diagnosis and treatment, cardiovascular diseases (CVDs) remain the leading cause of death in Europe [1]. One of the reasons for this issue is the rising prevalence of cardiovascular (CV) risk factors, subclinical atherosclerosis, and minimal effectiveness of prevention programs [1]. The growing population of young and middle-aged patients presenting with increased CV risk is particularly distressing. The increasing number of individuals with obesity, unrecognized hypertension, and metabolic abnormalities poses a problem [1–3].

It is widely accepted that professional soldiers should not have CVDs. However, even when carefully selected in terms of exercise and general health, they are not free from CV risk factors. Webber et al. [4] showed a 12.1% prevalence of atherosclerosis (coronary or aortic) in a cross-sectional study conducted among United States military service members who died in combat operations (*n* = 3832; mean age: 25.9 years; data from autopsy). In a study of 112 young Polish soldiers, Gielerak at al. [5] revealed that CV risk factors were present in over 50% of soldiers. Exposure to extensive stress and threats in combat conditions were suggested to have an additive detrimental effect [5].

Since most CV risk factors can be controlled, it is reasonable to focus on the identification of CV risk in a large population of Polish soldiers. For this reason, a preventive program for Polish armed forces personnel, the Equalization of Accessibility to Cardiology Prophylaxis and Care for Professional Soldiers, or MIL-SCORE, program was employed from 2013 to 2016. We aimed to present its main results and reveal the prevalence of CV risk factors in a population of Polish soldiers with respect to age.

## 2. Materials and Methods

### 2.1. Study Protocol and Study Population

The MIL-SCORE program was designed to assess the prevalence and management of CV risk factors as well as to establish a CVD monitoring system in the Polish armed forces. The program was announced by a decision from the Polish Ministry of Military Defense (decision no. 15/MON; February 12, 2013) and included approximately 5% of all professional soldiers (*n* = 6440). The program was coordinated by the Military Institute of Medicine (Warsaw, Poland) in cooperation with three medical centers (the 5th Military Clinical Hospital with Polyclinic in Krakow; the 10th Military Clinical Hospital with Polyclinic in Bydgoszcz; the Military Specialist Medical Clinic in Legionowo). Between 2013 and 2016, medical teams that included cardiac nurses and cardiologists visited selected military units. Soldiers who were absent at the time of the visit were encouraged to report personally to the nearest recruitment center.

The study protocol included a face-to-face medical history, physical examination, and laboratory tests. All data were documented in a detailed questionnaire form. All patients gave written informed consent to participate in the study.

### 2.2. Clinical Assessment

#### 2.2.1. Interview

Clinical assessment at the time of enrolment involved collection of the following sociodemographic data: age, gender, military rank, education level, and place of residence (i.e., countryside or town). The medical history focused on past and present chronic diseases including the following: hypertension (uncontrolled defined as a blood pressure (BP) >135/85 mmHg by out-of-office self-measurements), coronary artery disease, myocardial infarction, percutaneous transluminal coronary angioplasty, coronary artery bypass grafting, heart failure, peripheral arterial disease, chronic kidney disease, diabetes, and chronic obstructive pulmonary disease. A family history of CV risk factors and diseases was also investigated.

Physical activity was stratified as follows: (1) sedentary (i.e., sedentary lifestyle and occasional exercise), (2) moderate (i.e., regular vigorous activities less than 2.5 h per week), and (3) high (i.e., regular vigorous activities for more than 2.5 h per week). Smoking status categories were as follows: nonsmoker, former smoker, or current smoker.

#### 2.2.2. Physical Examination

The following parameters were evaluated during physical examination: weight (kg), height (cm), and body mass index (BMI, calculated as the weight in kilograms divided by the square of the height in meters). The heart rate (HR) and office systolic blood pressure (SBP) and diastolic blood pressure (DBP) were measured in a quiet room in the presence of a trained physician or nurse following a minimum of 5 min of rest in a seated position. The category of hypertension was defined according to the European Society of Cardiology (ESC) guidelines [6].

#### 2.2.3. Laboratory Tests

Blood samples were collected, and the concentrations of the following biochemical markers were determined using standard methods: total cholesterol (TC), triglycerides (TG), low-density lipoprotein cholesterol (LDL-C), high-density lipoprotein cholesterol (HDL-C), and glucose. All blood samples were processed at the field centers, immediately frozen, and transferred to the laboratories of the leading centers.

#### 2.2.4. Cardiovascular Risk Assessment

The 10-year CV risk was calculated using the SCORE Risk Chart that was recalibrated for the Polish population (Pol-SCORE 2015) [7]. Individuals who scored <1% were categorized as low risk; those who scored between 1% and 5% were categorized as moderate risk; those who scored between 5% and 10% were categorized as high risk; those who scored ≥10% were categorized as very high risk. Relative risk charts were used for CV risk assessment in individuals aged 20–40 years [1]. Individuals with at least one of the following factors were categorized as high or very high CV risk: documented CVD history, diabetes (with the exception of young people with type 1 diabetes and without major risk factors), markedly elevated cholesterol (>310 mg/dL) or BP (>180/110 mmHg), or chronic kidney disease (eGFR <60 mL/min/1.73 m^2^), according to European Guidelines on cardiovascular disease prevention [1].

### 2.3. Statistical Analysis

The quality of the data was critically evaluated, and data that were most likely erroneous were eliminated. In the final analysis, missing data for any variable did not exceed 5%, which should be considered a limitation that did not significantly affect the results. The numbers of complete records of the analyzed parameters are given in the tables.

The distribution and normality of the data were assessed via visual inspection and the Kolmogorov–Smirnov test. Continuous variables were presented as means ± standard deviations (SDs), and categorical variables were presented as absolute and relative frequencies (percentages). For comparative analysis, the study group was stratified by age as follows: 20 to 30 years (*n* = 2324); 31 to 40 years (*n* = 2471); 41 to 50 years (*n* = 1343); over 50 years (*n* = 302). These subgroups were compared with an ANOVA for continuous variables and the Chi-square test or Fisher's exact test for categorical variables. A two-tailed *p* value of <0.05 was considered statistically significant. Statistical analyses were performed using Statistica 12.0 (StatSoft, Inc., Tulsa, USA).

## 3. Results

A total of 6440 soldiers (mean age 34.9 ± 8.1 years; 97% male) participated in the study (Table 1). They represented the whole range of military ranks, with a predominance of privates, corporals, and noncommissioned officers. More soldiers lived in towns than in the countryside, and this difference was more strongly pronounced in soldiers over 50 years of age. The majority graduated from at least a secondary school. Almost half of the soldiers were past or current smokers, and the proportion of those who quit smoking increased with age. Occasional physical activity was reported by 12% of soldiers under 40 years of age and approximately 30% of those over 40 years of age (Table 1).

There were significant differences in BP among the subgroups, which demonstrated higher BP values in older soldiers. Among the entire study population, 14% of soldiers reported a history of hypertension.

The prevalence of hypertension increased with age, up to 44% in soldiers over 50 years of age (Table 2). Well-controlled home BP measurements were reported by only 40% of soldiers. In over 50% of soldiers, BP was abnormally high when measured in the office, and 14% of the values corresponded to grade 2 or 3 hypertension. Furthermore, one-third of normotensive soldiers had a high normal BP. The number of soldiers with increased BP when measured in the office was particularly high among soldiers who reported a history of hypertension (86%).

Over one-third of the total group was overweight. The prevalence of overweight and obese soldiers increased with age and reached 58% and 27%, respectively, in those over 50 years of age. Coronary artery disease was noted in 3% of soldiers over 50 years of age and <1% of younger soldiers. Diabetes affected 7% of the oldest soldiers and 3% of soldiers aged 40–50 years. The prevalence of other reported chronic diseases was marginal (<1%). The detailed results are presented in Table 2.

The detailed results of laboratory tests are presented in Table 3. TC was increased in over one-half of subjects. The proportion of those with abnormal LDL-C was even higher (60%). These results were accompanied by increased TG in 36% of soldiers, low HDL-C in 13% of soldiers, and glucose >100 mg/dl in 16% of soldiers. The prevalence of these disturbances increased with age, especially for LDL-C and glucose. Metabolic abnormalities and the high prevalence of hypertension were reflected in the results of CV risk assessment (Table 4). Out of a total of 5985 soldiers, those with a calculated CV risk of 3% (*n* = 189) were automatically categorized as high or very high CV risk. The others, most of whom were 40–50 years of age, were classified as moderate CV risk. In a subgroup of soldiers >50 years of age, high and very high CV risk scores were designated for almost one-third of soldiers. The relative risk assessed in younger subgroups (<40 years of age) was moderate or high.

Subanalysis of soldiers 20–30 years of age revealed that predominant office work (more than 75% of time spent at office) was not linked with any significant differences in cardiovascular risk factors compared with soldiers physically active at work. Major difference was in smoking habit, which was less prevalent in soldiers with predominant office work (23.2% vs. 33.9%; *p*=0.01). The differences were seen in almost all cardiovascular risk factors when comparing soldiers exercising sporadically with soldiers regularly active (waist circumference, BMI, LDL and HDL cholesterol, blood pressure measurements, and less smoking). Interestingly, prevalence of hypertension was comparable in both groups (6.5 vs. 5.1%, *p*=0.38).

**Figure 1:**
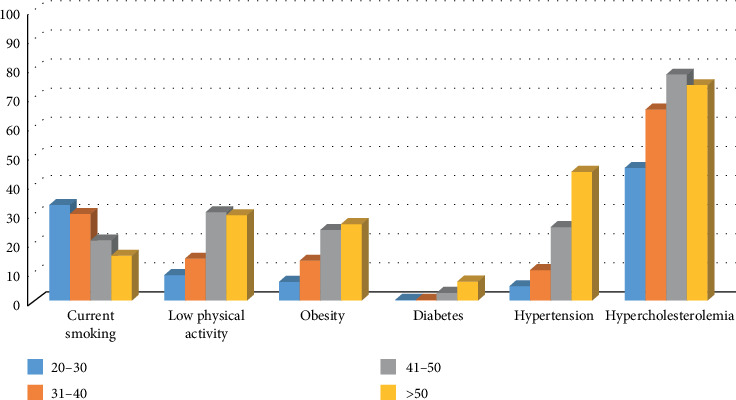
Distribution of risk factors divided by age (age groups: 20–30, 31–40, 41–50 and >50 years of age). Scale on the left shows percentage of soldiers with risk factor. Obesity: BMI >30 kg/m^2^; hypercholesterolemia: LDL cholesterol >115 mg/dl.

Summary of the prevalence of the risk factors is shown on Figure 1.

## 4. Discussion

The results of this program indicate that the cardiovascular conditions of professional Polish soldiers are consistent with the unfavorable trends in the general population. The high prevalence of obesity, metabolic disorders, and abnormal blood pressure require special attention. The incidence of CV risk factors increases with age, and the synergistic effect of these risk factors translates into an alarming number of individuals at risk of atherosclerotic events.

The program included approximately 5% of all Polish professional soldiers, which were mainly men (97%). The sample size was similar to that in the WOBASZ II study (6440 vs. 6163) [8]. Most of the soldiers were young (mean age 35 years), and only 26% reached the fifth and sixth decade of life. Although the number of current smokers decreased with age, a high percentage of smokers was observed (46%). A negative age-related trend in physical activity was noted. Among soldiers over 40 years of age, almost 30% reported a sedentary lifestyle. Since smoking and physical inactivity are strong predictors of CV events [1], lifestyle modifications should be first-line treatment in this population.

The results of office BP measurements suggest a higher prevalence of hypertension than was reported by the soldiers. Only 32% of soldiers had an office BP <130/80 mmHg; 16% had high normal values, and 52% had hypertension (>140/90 mmHg). In the WOBASZ II study [8], optimal BP was observed in 34.2% of men and 22% of those aged 19–49 years. Compared with age-paired groups, the mean BP in the WOBASZ study was slightly lower than that in our study [8, 9].

In our study population, the number of soldiers with previously diagnosed hypertension increased with age. Less than half of hypertensive soldiers reported good BP control according to home BP measurements (39%). The results of office measurements were even worse; no more than 14% of hypertensive soldiers had a BP <140/90 mmHg. These observations are consistent with the NATPOL PLUS results, in which effective hypertensive treatment was noted in only 12% of participants [10]. In the WOBASZ II study, BP normalization was reported in 19% of men. The authors of the WOBASZ II study observed the worst pressure control in young participants (i.e., those aged 19–49 years) [8].

Most soldiers were overweight (55%) or obese (14%). The high prevalence of obesity correlated with an age-related negative trend in physical activity. Among young soldiers, only 10% were obese. However, less than 50% of them had a normal BMI. One-fourth of soldiers aged over 40 years had a BMI >30 kg/m2. Abnormal weight was reported in 62% of participants in the WOBASZ study [8], and 53% of participants in the NATPOL PLUS study [10].

Metabolic abnormalities were represented by high LDL-C in 60% of soldiers, high TG in 36% of soldiers, and high TC in 52% of soldiers. These results are comparable with those in previous reports. Gielerak et al. [5] reported dyslipidemia in 51% of young (mean age 26.9 ± 4.3 years), healthy male soldiers (*n* = 112). Mazurek et al. [11] revealed hypercholesterolemia in 72.4% of 272 Polish military pilots. In a population of Polish professional drivers (96% men, mean age 50 years, mean BMI 32 kg/m^2^), Krzowski et al. [12] noted hypercholesterolemia in 50% of participants, high LDL-C in 72% of participants, and high TG in 29% of participants. Only low HDL-C was significantly higher (84%) than in our study group (13%) [11]. In the NATPOL 2011 study [10], hypercholesterolemia was reported in 61% of participants, and hypertriglyceridemia was reported in 21% of participants. A slightly higher prevalence of dyslipidemia was reported in the WOBASZ study (69% in women and 74% in men) [9, 13].

The prevalence of previously diagnosed diabetes in the entire study population was low (1%). However, among soldiers over 50 years of age, its prevalence reached 7%, which was similar to that of the general population [14]. The plasma glucose concentration exceeded 100 mg/dl in 16% of soldiers and almost half of soldiers over 50 years of age. Although nonfasting glucose measurements might overestimate the prevalence of increased plasma glucose concentrations, the true incidence of carbohydrate disorders should be verified more thoroughly.

Due to the aforementioned CV risk factor data, it is not surprising that the majority of subjects were categorized as moderate or high CV risk. The low prevalence of low CV risk (*n* = 9) among soldiers, a population that is expected to be notably healthy, is distressing.

A high prevalence of CV risk factors was also reported in the military personnel of other nations. O'Donnell et al. [15] performed an extensive analysis of the data from the Defense Medical Surveillance System records for 3,105,061 active duty soldiers who served in the United States Armed Forces at any time between 2007 and 2016. During that period, 18% of all service members were diagnosed with at least one of the following CV risk factors: hypertension, hyperlipidemia, obesity, or abnormal blood glucose. In an age subgroup analysis, the prevalence of obesity increased with age and reached a rate of 23.5 per 1000 person-years in those aged over 50 years and 18.3 in those aged 20–29 years. The same trend was observed for hyperlipidemia (80.6 versus 9.2, respectively), abnormal glucose levels (28.9 versus 2.0, respectively), and hypertension (54.4 versus 10.0, respectively). Therefore, many American soldiers have developed or have been discovered to have CV risk factors while in the military service despite being more physically active and in good physical health at the time of enlistment in the army. The CV risk factor prevalence is higher in military healthcare members than in those of other occupations.

Grosz et al. [16] reported a prevalence of 41% for obesity, 32% for smoking, 24% for physical inactivity, and 54% for hypercholesterolemia among 250 male Hungarian military pilots. The only significant difference from our population was the low incidence of hypertension (15%). Among Lithuanian active duty military personnel (*n* = 200, 126 men), hypercholesterolemia was discovered in 45% of the men, and its prevalence increased with age; in participants aged 45–54 years, it was 74% [17]. The proportion of current male smokers was also high (46%) and coexisted with metabolic disturbances [17]. In young male Brazilian military personnel (*n* = 380, aged 19–35 years), Wenzel et al. [18] revealed that smoking was associated with the prevalence of hypertension (22%), and a lack of regular physical activity was also associated with the prevalence of hypertension.

We demonstrated that in the 20–30 years of age group the absolute number of cardiovascular factors is very low. Hypertension was present in 5% of soldiers, compared with about 10% of soldiers of that age in the American group. Diabetes was not prevalent in any of the group (0% of our group, 0.3% of American soldiers) [15]. The other authors have shown that among young adults aged 18–39 years 8.8% had hypercholesterolemia and 7.3% had hypertension [19]. Although we do not have data on the cardiovascular risk factors, it is worth to notice that severe coronary stenosis was found in about 15% of soldiers who died in Korean war in autopsy studies and in 5% of soldiers in autopsy studies of Vietnam war victims. In contemporary autopsy studies, the rate of severe atherosclerosis was 2.3% in soldiers predominantly below 30 years old [4]. The most probable reason for that decrease is a significant drop in smoking prevalence [1]although when looking at the trends over the last 40 years in the whole European population there is also a decrease in mean blood pressure and mean cholesterol level, but an increase in mean BMI and in prevalence of diabetes (data from 1980 to 2012–2015). [20].

Our results confirm that young adults who are physically active have less cardiovascular risk factors. The same was shown in Cardia study (young adults aged 18–30 years). Healthy lifestyle factors (i.e., not currently smoking, BMI < 25, and being on a healthy diet) were inversely related to total cholesterol, blood pressure, BMI, triglycerides, lipids, and fasting glucose [21]. This is consistent with cardiovascular prevention guidelines [1].

### 4.1. Clinical Implications

Due to the diagnosed health problems of Polish professional soldiers, it seems necessary to implement a comprehensive system that would include elements of effective prevention, diagnosis, and optimal treatment of CV risk factors. Such an adequate approach can reduce the likelihood of significant CVDs not only for those serving the military but also for those who have left the armed forces. Further research should focus on the identification of CV risk factors specific to military service personnel. Other occupational groups may also benefit from such an approach.

### 4.2. Limitations

One of the limitations is to some extent lack of originality of the results; there are publications showing distribution of the cardiovascular risk factors in different populations. The main limitation of our methodology was that the examined group included volunteers and was not a stratified random sample. Moreover, the population was dominated by men, which was due to the demographic structure of the Polish armed forces. BP measurement at only one visit might have resulted in the overestimation of hypertension prevalence. According to previous data, this overestimation affects 12.6% of participants [22]. Nonfasting laboratory tests also require caution in their interpretation. Detailed data concerning medications was not collected.

## 5. Conclusions

Soldiers are regarded as the epitome of health and fitness. However, findings from this study suggest that this occupational group does indeed have multiple CV risk factors and mirrors trends seen in the general population. Preventive programs aimed at early CV risk assessment and modification, including smoking cessation and management of hypertension and nutritional and metabolic disturbances, are strongly needed in this population.

## Figures and Tables

**Table 1:** Sociodemographic data.

	Total	20–30 years of age	31–40 years of age	41–50 years of age	>50 years of age	*p* value
Gender (male/female)^6440^	6245/195 (97.0/3.0)	2237/87 (96.3/3.7)	2384/87 (96.5/3.5)	1325/18 (98.7/1/3)	299/3 (99.0/1.0)	0.00003
Age (years)^6440^	34.9 ± 8.1	26.8 ± 2.4	35.2 ± 2.9	44.3 ± 2.8	54.0 ± 3.1	
Residence (countryside/town)^6169^	1788/4381 (30.0/70.0)	798/1460 (35.3/64.6)	703/1692 (29.4/70.6)	252/1008 (20.0/80.0)	35/221 (13.7/86.3)	<0.00001
*Family history* ^*6333*^						
Coronary artery disease	732 (11.6)	126 (5.4)	268 (10.9)	273 (21.1)	65 (24.4)	<0.00001
Hypertension	1302 (20.6)	332 (14.3)	541 (22.1)	348 (26.8)	81 (30.5)	<0.00001
Stroke	275 (4.3)	46 (2.0)	112 (4.6)	90 (6.9)	27 (10.2)	<0.00001
Diabetes	695 (11.0)	157 (6.8)	281 (11.5)	224 (17.3)	33 (12.4)	<0.00001
*Smoking status* ^*6244*^						
Yes/current^*∗*^	2933 (46.2)/1844 (62.9)	1069 (46.8)/772 (72.2)	1149 (47.3)/743 (64.7)	551 (43.1)/282 (51.1)	113 (44.5)/47 (41.6)	0.075/<0.00001
Smoking: pack-years (median, interquartile range)	6 (2.5–11.25)	4 (1.6–7.0)	7 (3.0–12.0)	11 (5.0–20.0)	18.9 (10.0–30.0)	<0.0001
*Physical activity* ^*6311*^						
Occasional	1051 (16.7)	213 (9.2)	361 (14.8)	400 (30.9)	77 (29.5)	<0.00001
Moderate	2773 (43.9)	1307 (56.5)	1068 (43.7)	335 (25.9)	63 (24.1)	
High	2487 (39.4)	793 (34.3)	1015 (41.5)	558 (43.2)	121 (46.4)	

Data presented as mean ± standard deviation (SD) and number (percentage), respectively; ^*∗*^Among smokers responding yes.

**Table 2:** Basic clinical characteristics.

	Total	20–30 years of age	31–40 years of age	41–50 years of age	>50 years of age	*p* value
HR (bpm)^6311^	73.7 ± 11.8	74.0 ± 11.7	73.9 ± 12.0	73.2 ± 11.3	72.2 ± 12.2	0.033
SBP (mmHg)^6304^	134.7 ± 16.5	132.7 ± 15.8	134.8 ± 16.2	137.7 ± 17.6	137.4 ± 18.5	<0.00001
DBP (mmHg)^6304^	83.1 ± 11.4	79.5 ± 10.6	83.6 ± 10.8	87.4 ± 10.8	88.0 ± 12.4	<0.00001
BP = 130–139/85–89 (mmHg)^6304^	1006 (16.0)	358 (15.5)	378 (15.5)	225 (17.4)	45 (17.1)	<0.00001
BP = 140–159/90–99 (mmHg)^6304^	2403 (38.1)	754 (32.6)	1003 (41.1)	535 (41.4)	111 (42.2)	<0.00001
BP = 160–179/100–109 (mmHg)^6304^	708 (11.2)	163 (7.0)	268 (11.0)	222 (17.2)	55 (20.9)	<0.00001
BP > 180/110 (mmHg)^6304^	158 (2.5)	27 (1.2)	55 (2.2)	63 (4.9)	13 (4.9)	<0.00001
BMI (kg/m^2^)^6316^	26.7 ± 3.3	25.6 ± 2.9	26.8 ± 3.1	28.0 ± 3.5	28.2 ± 3.3	<0.00001
Overweight (BMI 25–29.9 kg/m^2^)^6316^	3447 (54.6)	1152 (49.8)	1407 (57.5)	735 (56.9)	153 (58.2)	<0.00001
Obesity (BMI ≥30.0 kg/m^2^)^6316^	893 (14.1)	157 (6.8)	345 (14.1)	320 (24.8)	71 (27.0)	<0.00001
*Chronic diseases in anamnesis* ^*6333*^						
Coronary artery disease	14 (0.2)	0 (0.0)	0 (0.0)	5 (0.4)	9 (3.4)	<0.00001
Myocardial infarction	10 (0.2)	0 (0.0)	0 (0.0)	7 (0.5)	3 (1.1)	<0.00001
Chronic kidney disease	11 (0.2)	1 (0.04)	5 (0.2)	5 (0.4)	0 (0.0)	0.101
Diabetes mellitus	63 (1.0)	0 (0.0)	6 (0.24)	39 (3.0)	18 (6.8)	<0.00001
Chronic obstructive pulmonary disease	10 (0.2)	0 (0.0)	4 (0.2)	4 (0.3)	2 (0.8)	0.009
Hypertension	834 (13.7)	122 (5.3)	260 (10.6)	333 (25.7)	1119 (44.7)	<0.00001
Hypertension in medical history and office BP ≥140/90 mmHg	717 (86.0)	90 (73.8)	237 (91.1)	292 (88.0)	98 (82.4)	—
Declared well home BP control: confirmed well BP control^758^	293 (38.7)/465 (61.3)	24 (23.8)/77 (76.2)	67 (28.3)/170 (71.7)	145 (47.2)/162 (52.8)	57 (50.4)/56 (49.6)	<0.00001

Data presented as mean ± standard deviation (SD) and number (percentage) ^*∗*^*p* value not calculated (very low prevalence). BP: blood pressure; DBP: diastolic blood pressure; BMI: body mass index; HR: heart rate; SBP: systolic blood pressure.

**Table 3:** The results of laboratory tests.

	Total	20–30 years of age	31–40 years of age	41–50 years of age	>50 years of age	*p* value
Glucose (mg/dl)^6255^	88.8 ± 23.2	83.8 ± 20.7	88.3 ± 17.3	95.2 ± 23.8	106.1 ± 53.7	<0.00001
TC (mg/dl)^6440^	197.5 ± 42.0	181.5 ± 37.0	202.0 ± 39.2	215.1 ± 44.2	211.4 ± 48.4	<0.00001
TG (mg/dl)^6285^	152.0 ± 121.3	128.3 ± 88.2	158.7 ± 117.9	180.9 ± 167.7	156.5 ± 91.6	<0.00001
LDL-C (mg/dl)^6440^	128.3 ± 36.8	114.6 ± 32.2	131.8 ± 35.1	144.3 ± 38.5	141.6 ± 38.5	<0.00001
HDL-C (mg/dl)^6262^	52.7 ± 12.7	53.4 ± 12.5	52.7 ± 13.0	51.6 ± 12.6	52.0 ± 11.5	0.0007
Glucose >100 mg/dl^6255^	993 (15.9)	189 (8.3)	366 (15.0)	322 (25.2)	116 (45.3)	<0.00001
T-C >190 mg/dl^6440^	3357 (52.1)	820 (35.3)	1430 (57.9)	937 (69.8)	170 (56.3)	<0.00001
LDL-C >115 mg/dl^6245^	3844 (59.8)	1064 (46.1)	1604 (66.3)	989 (78.2)	187 (74.2)	<0.00001
TG >150 mg/dl^6285^	2274 (36.2)	606 (26.3)	955 (39.1)	608 (47.4)	105 (40.7)	<0.00001
HDL-C <40 mg/dl (men) and <46 mg/dl (women)^6262^	823 (13.1)	262 (11.4)	328 (13.5)	200 (15.7)	33 (12.9)	0.003

Data presented as mean ± standard deviation (SD) and number (percentage). HDL: high density lipoproteins cholesterol; LDL-C: low density lipoproteins cholesterol; TC: total cholesterol; TG: triglycerides.

**Table 4:** Cardiovascular risk assessment and selected single risk factors by age.

	Total	20–30 years of age	31–40 years of age	41–50 years of age	>50 years of age	*p* value
*CV risk by SCORE* ^*5985*^						
Mean SCORE	2.4 ± 1.5	2.4 ± 1.3	2.7 ± 1.5	1.5 ± 1.0	4.3 ± 2.5	<0.00001
SCORE < 1%	9 (0.2)^*∗*^	0 (0.0)^*∗*^	0 (0.0)	9 (0.8)	0 (0.0)	<0.00001
SCORE ≥1 and <5%	5479 (91.5)^*∗*^	2080 (93.0)^*∗*^	2091 (89.5)	1161 (97.5)	147 (66.5)	
SCORE ≥5% and <10%	472 (7.9)^*∗*^	155 (6.9)^*∗*^	237 (10.2)	18 (1.5)	62 (28.1)	
SCORE ≥10%	25 (0.4)^*∗*^	2 (0.1)^*∗*^	8 (0.3)	3 (0.3)	12 (5.4)	
*Prevalence of single clinical markers of high or very high CV risk*						
BP >180/110 mmHg^6304^	158 (2.5)	27 (1.2)	55 (2.2)	63 (4.9)	13 (4.9)	<0.00001
T-C >310 mg/dl^6440^	89 (1.4)	8 (0.3)	37 (1.5)	35 (2.6)	9 (3.0)	<0.00001
Coronary artery disease^6333^	14 (0.2)	0 (0.0)	0 (0.0)	5 (0.4)	9 (3.4)	<0.00001
Chronic kidney disease^6333^	11 (0.2)	1 (0.04)	5 (0.2)	5 (0.4)	0 (0.0)	0.101
Diabetes mellitus^6333^	63 (1.0)	0 (0.0)	6 (0.24)	39 (3.0)	18 (6.8)	<0.00001

Data presented as number (percentage). ^*∗*^Relative risk. BP: blood pressure; SCORE: systematic coronary risk evaluation.

## Data Availability

The data used to support the findings of this study are available from the corresponding author upon request.
